# Factors influencing the levothyroxine dose in the hormone replacement therapy of primary hypothyroidism in adults

**DOI:** 10.1007/s11154-021-09691-9

**Published:** 2021-10-20

**Authors:** Philippe Caron, Solange Grunenwald, Luca Persani, Françoise Borson-Chazot, Remy Leroy, Leonidas Duntas

**Affiliations:** 1 Service d’Endocrinologie, Maladies métaboliques et Nutrition, Hôpital Larrey, CHU de Toulouse, 24 chemin de Pouvourville, 31059 Toulouse Cedex, France; 2 Department of Medical Biotechnologies and Translational Medicine, University of Milan, Milan, Italy; 3 Division of Endocrine and Metabolic Diseases, IRCCS Istituto Auxologico Italiano, Milan, Italy; 4 Fédération d’Endocrinologie, Hôpital Louis Pradel, Hospices Civils de Lyon, Bron, France; 5 Research on Healthcare Performance (RESHAPE), INSERM U1290, Université Claude Bernard Lyon 1, Lyon, France; 6 Private Office, Lille, France; 7 Unit of Endocrinology, Diabetes and Metabolism Division, Evgenideion Hospital, University of Athens, Athens, Greece

**Keywords:** Absorption, Metabolism, Deiodinases, Drugs, LT4 absorption test, Pseudomalabsorption

## Abstract

Levothyroxine (LT4) is a safe, effective means of hormone replacement therapy for hypothyroidism. Here, we review the pharmaceutical, pathophysiological and behavioural factors influencing the absorption, distribution, metabolism and excretion of LT4. Any factor that alters the state of the epithelium in the stomach or small intestine will reduce and/or slow absorption of LT4; these include ulcerative colitis, coeliac disease, bariatric surgery, *Helicobacter pylori* infection, food intolerance, gastritis, mineral supplements, dietary fibre, resins, and various drugs. Once in the circulation, LT4 is almost fully bound to plasma proteins. Although free T4 (FT4) and liothyronine concentrations are extensively buffered, it is possible that drug- or disorder-induced changes in plasma proteins levels can modify free hormone levels. The data on the clinical significance of genetic variants in deiodinase genes are contradictory, and wide-scale genotyping of hypothyroid patients is not currently justified. We developed a decision tree for the physician faced with an abnormally high thyroid-stimulating hormone (TSH) level in a patient reporting adequate compliance with the recommended LT4 dose. The physician should review medications, the medical history and the serum FT4 level and check for acute adrenal insufficiency, heterophilic anti-TSH antibodies, antibodies against gastric and intestinal components (gastric parietal cells, endomysium, and tissue transglutaminase 2), and *Helicobacter pylori* infection. The next step is an LT4 pharmacodynamic absorption test; poor LT4 absorption should prompt a consultation with a gastroenterologist and (depending on the findings) an increase in the LT4 dose level. An in-depth etiological investigation can reveal visceral disorders and, especially, digestive tract disorders.

## Introduction

The drug levothyroxine (LT4) constitutes a safe, effective hormone replacement therapy for hypothyroidism caused by autoimmune thyroiditis, partial or total thyroidectomy, radioiodine treatment, or drug side effects [1–5]. LT4 is primarily given *per os* as a tablet, soft-gel or liquid (drop) formulation, although liquid preparations for intramuscular or intravenous injection are also available. The chronic nature and high prevalence of hypothyroidism (~4% of adults in Western countries), together with the unaltered life expectancy of treated patients, explain why LT4 is one of the world’s most extensively prescribed drugs [6–9]. However, LT4 has a narrow therapeutic window, and around 30% of patients fail to stably achieve the recommended serum level of thyroid-stimulating hormone (TSH; between 0.4 and 4.0 mIU/L, according to the American Thyroid Association guidelines) with a standard, body-weight-based starting dose of LT4 (typically 1.5 to 1.7 µg/kg/day in patients with residual endogenous thyroid function, such as those with auto-immune thyroiditis) [2, 10–12]. The dose of LT4 required by a patient can be predicted from the total body weight, the body mass index (BMI), ideal body weight, and lean body mass, with the latter three parameters providing the most accurate estimates. Several equations have been developed to calculate the dose requirement [13].

Hence, for each individual patient with hypothyroidism, the challenge for the clinician is to find the oral dose of LT4 that relieves the clinical signs and symptoms of hypothyroidism a condition that is typically associated with the restoration of parameters of thyroid hormone (TH) action (primarily the serum TSH level) to within the age-appropriate physiological reference (or target) range.

**Fig. 1:**
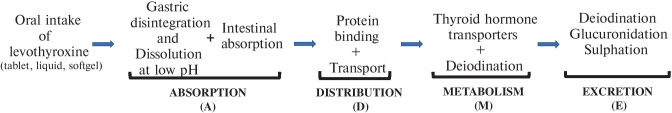
An overview of ADME processes for orally administered LT4 in the treatment of hypothyroidism

**Fig. 2:**
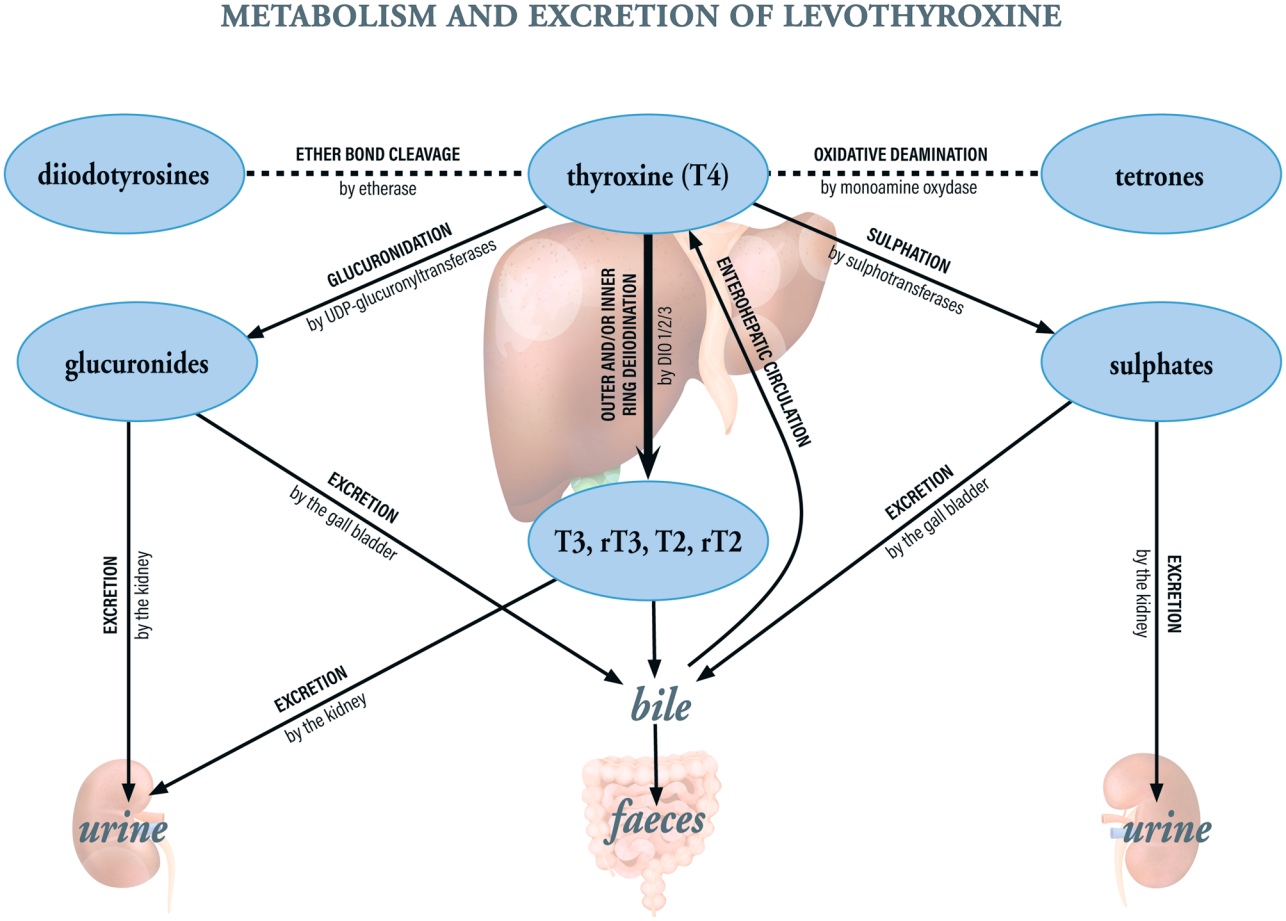
Pathways and sites for the metabolism and excretion of LT4

The clinical effectiveness of any drug depends on pharmaceutical, pathophysiological (internal) and behavioural (external) factors. Many of these factors interfere with absorption, distribution, metabolism and excretion (ADME). The overall ADME process for LT4 is summarized in Fig. 1. Oral LT4 (typically formulated as the sodium salt) dissolves in the stomach at low pH but is absorbed mainly in the small intestine (the jejunum and ilium) within three hours of ingestion [14, 15]. The bioavailability of LT4 is 65–80% in fasting euthyroid subjects and hypothyroid patients [16, 17]. Once the LT4 has crossed the intestinal epithelium and reached the circulation, it is almost entirely (~99.9%) bound to plasma proteins (mainly albumin, thyroxine-binding globulin (TBG), transthyretin, and high-density lipoprotein). The mean distribution volume of LT4 is 11–12 L in euthyroid subjects and (due to fluid retention) up to 15 L in hypothyroid patients [18, 19]. T4 is metabolized in several organs, glands, and areas of the brain (mainly the liver but also the thyroid, anterior pituitary, and kidney) and in peripheral tissues (e.g. the muscles) (Fig. 2) [20]. Primarily, deiodination of T4’s outer ring by type 2 deiodinase (DIO2) gives liothyronine (L-tri-iodothyronine or T3, the main biologically active metabolite), and deiodination of the inner ring by type 1 or type 3 deiodinases (DIO1 or DIO3) gives the biologically inactive reverse T3 (rT3) [21]. Around 20% of the ingested dose of LT4 is ultimately eliminated in the stools; this includes T4 and T3 excreted in the bile after glucuronidation or sulphation in the enterohepatic cycle (Fig. 2) [20]. The remaining 80% is excreted in the urine [20]. The half-life of T4 is reportedly 6–7 days in euthyroid subjects and 7–8 days in hypothyroid patients [22]. However, the relationships between levels of TSH, free T4 (FT4) and free T3 (FT3) are not the same in LT4-treated patients as in healthy, euthyroid individuals, and the distribution, metabolism and excretion of exogenous LT4 differ from those of endogenous T4 [23].

When faced with insufficient initial effectiveness of LT4 or a loss of effectiveness in a previously stable patient, the physician will typically consider increasing the daily dose. However, it is at this moment that the physician should ask him/herself whether LT4’s apparent lack of effectiveness is related to one or more factors that interfere with the drug’s ADME. Of course, one must always seek to distinguish between true malabsorption and pseudomalabsorption; the latter is usually due to poor treatment compliance (i.e. not taking the prescribed LT4). A four- to six-hour LT4 absorption test (i.e. the monitoring of blood T4 levels after the supervised administration of a weight-based dose of LT4) is the mainstay of this distinction [24]. The paracetamol absorption test has also been used as control; given that paracetamol and LT4 differ in their physicochemical properties and absorption mechanisms, malabsorption of both will only be caused by severe dysfunction of the gastrointestinal tract, and so an abnormal LT4 absorption test and a normal paracetamol test is suggestive of poor compliance with LT4 treatment [25].

Hence, the primary objective of the present review was to draw up a comprehensive list of pharmaceutical, pathophysiological (internal) and behavioural (external) factors that influence the absorption, distribution, metabolism and excretion of LT4 and can therefore condition the drug’s effectiveness, safety and required dose. The secondary objective was to provide practical decision support for physicians managing patients with “treatment-refractory” hypothyroidism, unexpectedly poor effectiveness of LT4, or suspected LT4 malabsorption.

## Methods

### Literature search and analysis

We searched the PubMed database from January 1^st^, 2000, to April 30^th^, 2021, with logical combinations of the following terms (in English only): levothyrox*, LT4, L-T4, liothyronin*, T3, tetraiodothyro*, triiodothyro*, absorp*, absorb*, distrib*, transport*, metabol*, eliminat*, excret*, deiodinase*, bioavailab*, titrat*, food*, interacti*, and enterohepat*. For example, a sample search was (levothyrox* OR LT4 OR L-T4 OR liothyronin* OR T3 OR tetraiodothyro* OR triiodothyro*) AND (absorp* OR absorb* OR malabsorb* OR pseudomalabsorb* OR distrib* OR transport* OR metabol* OR eliminat* OR excret* OR bioavailab* OR titrat*) AND hypothyro* NOT rat NOT mouse NOT chicken NOT zebrafish), with the filters Clinical Study, Clinical Trial, Clinical Trial, Phase I, Clinical Trial, Phase II, Clinical Trial, Phase III, Clinical Trial, Phase IV, Comparative Study, Controlled Clinical Trial, Multicenter Study, Observational Study, Pragmatic Clinical Trial, Randomized Controlled Trial, and Humans.

Abstracts were screened for relevance. If an abstract was found to be relevant, the full text article was retrieved and reviewed. We excluded case reports, reviews, and editorials. We arbitrarily classified factors influencing the relationship between the prescribed oral dose of LT4 and the drug’s clinical effectiveness into three broad categories: pharmaceutical factors (the dosing regimen, the time of day taken, the administration route, the pharmaceutical formulation), pathophysiological factors (aetiology, type of hypothyroidism, disease status, concomitant diseases or comorbidities, anthropometric variables, genetic variants, etc.), and behavioural (concomitant drug treatments, foods, compliance, the prescribing physician’s characteristics, etc.) (Table 1). The present study did not involve human subjects and therefore did not require review and approval by an institutional review board.

**Table 1:** Classification of factors influencing the effectiveness of LT4 and thus the dose required to achieve the target serum TSH level

Type of factor	Examples
Pharmaceutical	• Pharmaceutical formulation (tablet, gel, liquid, excipients, storage conditions, etc.)
	• Administration route (oral, intravenous, or intramuscular)
	• Dosing regimen (dosing frequency, time of day, before or after a meal, etc.)
	• Concomitant administration of other thyroid hormones (e.g. LT4 + LT3 combination therapy)
Pathophysiological (internal)	• Thyroid disorder (type, degree, and progression) and etiology (auto-immune disease, thyroid surgery, radioiodine treatment, etc.)
	• Comorbidities (type, degree, and progression)
	• Age, sex, body mass index, pregnancy, etc
	• Genetic variants (in the genes coding for deiodinases, TH transporters or receptors, for example) or possible acquired changes in TH action (a difficult TSH normalization is reported in some patients with congenital hypothyroidism)
	• Malabsorption
	• Changes in the underlying residual thyroid function
Behavioral (external)	• Concomitant intake of medications, foodstuffs, and food supplements
	• Poor compliance, pseudomalabsorption, and poor quality of life
	• Characteristics of the prescribing physician (medical specialty, country of practice, etc.)

*LT4* levothyroxine, *LT3* L-tri-iodothyronine, *TH* thyroid hormone, *TSH* thyroid-stimulating hormone

## Results

Our search initially identified a total of 741 potentially relevant publications, of which 140 were retrieved and 97 were reviewed. The main reasons for exclusion were animal studies (despite the “Human” and “Clinical Trial” filters in PubMed), case reports, and a lack of data on changes in LT4 ADME.

### Factors that interfere with the absorption of LT4

#### Pharmaceutical factors

Several studies have examined the time of day of LT4 intake (independently of food intake). Even though the summaries of product characteristics for LT4 formulations recommend intake on an empty stomach (typically on awakening, and at least 30 min before breakfast), patients are tempted to take their LT4 with (or only shortly before) their breakfast. In this respect, Guglielmi et al. open-label longitudinal study found that taking a liquid LT4 formulation with breakfast (rather than taking LT4 tablets on an empty stomach before breakfast) was associated with greater patient satisfaction, as judged by significant (*p* < 0.01) improvements in three of the seven subscores in the Underactive Thyroid Treatment Satisfaction Questionnaire [26].

The data on the influence of time of day are inconsistent – at least at first sight. Several studies did not observe an effect. For example, Skelin et al. did not observe significant differences in TSH, FT4 and FT3 levels for three times of day (30 min before breakfast, 60 min before lunch, and at least 120 min after an evening meal) for LT4 tablets [27], and Pirola et al. study of 4 hypothyroid patients treated with liquid LT4 did not find any significant differences in the serum TSH level according to whether the LT4 was taken 30 min before breakfast or at breakfast [28]. Similar findings were reported for serum TSH, FT4 and/or FT3 levels crossover trials [29–31].

In contrast, the results of several crossover studies argued in favour of administration before bedtime, after the evening meal is no longer in the stomach. Srivastava et al. observed significantly lower TSH levels and significantly higher FT3 and FT4 levels than with an equivalent morning dose regimen [32]. Bach-Huynh et al. three-period crossover study of 65 LT4-treated stable hypothyroid patients found that non-fasting LT4 administration was associated with higher and more variable serum TSH concentrations [33]. Lastly, Silva Perez et al. performed a prospective, randomized, open-label, crossover study of LT4 administration on an empty stomach for 90 days vs. LT4 with breakfast for 90 days. The TSH level was higher in the breakfast group than in the fasting group (2.89 vs. 1.9 mIU/L, *p* = 0.028) [34]. Lastly, Bolk et al. reported that administration of LT4 at bedtime was associated with higher thyroid hormone concentrations and lower TSH concentrations than administration in the morning [35].

Although circadian rhythms may influence LT4 administration [35], we conclude the main variability factor in the time of day is whether or not the stomach contains food or other ingested substances when oral LT4 is administered. Our clinical experience suggests that many patients are unwilling to fast in the morning; hence, LT4 might be more reliably taken at bedtime (i.e. at least 3 to 4 h after the evening meal).

The pharmaceutical formulation of LT4 can interact with factors that modulate the drug’s absorption [36]. In a randomized, single-dose, crossover pharmacokinetic bioequivalence study of 84 healthy subjects, three different orally administered dose-equivalent LT4 formulations (liquid, tablets, and soft-gel capsules) were found to have similar overall exposure rates and area under the concentration–time [37]. The time to peak was shortest (by around 30 min) for the liquid solution – prompting the researchers to suggest that this might reduce drug-LT4 interactions. Other reports from both retrospective and prospective observational studies indicate that orally administered liquid and soft-gel formulations of LT4 are associated with higher serum FT4 levels and lower TSH levels than tablet formulations, even when the overall LT4 dose level is the same – perhaps due to the absence of gastric disintegration [38–44]. Hence, liquid formulations might enable better TSH control and fewer LT4 dose adjustments. A liquid formulation might also reduce the likelihood of drug-drug or drug-supplement interactions. For example, Guglielmi et al. retrospective, real-world analysis of 3965 LT4-treated patients in Italy found that LT4 dose increases before and after a drug-drug indication (mainly due to proton pomp inhibitors and calcium or iron salts) were less likely in individuals taking a liquid LT4 formulation [45]. Furthermore, TSH variability was lower in patients taking the liquid LT4 formulation [45]. However, Benvenga and Di Bari observed abnormally high TSH levels in patients with inappropriate (undiluted) oral administration of liquid LT4—showing that use of a liquid formulation is not a panacea [46].

Lastly, the LT4 dosing frequency has rarely been investigated. In a randomized, crossover study of 14 patients. Bornschein reported that weekly oral LT4 administration (as a possible means of increasing treatment compliance) was associated with transient increases in FT4 levels and (after 6 weeks) a slight decrease in FT3 levels. There were no thyrotoxic symptoms or signs of cardiac problems [47].

In summary, the pharmaceutical formulation of LT4 and the time of day and/or frequency of LT4 administration are easily modifiable parameters that are worth testing when confronted with a potential case of malabsorption.

#### Pathophysiological factors

It is now clear that a high proportion of LT4-treated patients have a disease or condition (gastroesophageal reflux disease, irritated bowel syndrome, food allergies, lactose intolerance, gastric bypass, *H. pylori* infection, gastroparesis, coeliac disease, ulcerative colitis, Crohn’s disease, atrophic gastritis, etc.) that can reduce the intestinal absorption of LT4 [24, 48]. To reach an equivalent TSH level, the LT4 dose must be higher if the patient’s intestinal tract is affected by ulcerative colitis [49], coeliac disease [50], lactose intolerance [51], and gastritis (especially *Helicobacter pylori*–related gastritis and autoimmune gastritis) [43, 52–54], and infections with *Giardia lamblia* (giardiasis) [55]. In patients with gastritis, a change in LT4 formulation may be beneficial. For example, the administration of a liquid LT4 formulation in patients with hypothyroidism and an ongoing *Helicobacter pylori* infection was associated with a significantly greater fall in TSH levels, relative to administration of a tablet formulation [43].

Digestive tract surgery in general and bariatric surgery in particular lead to a reduction in BMI, which in turn should prompt a reduction in the LT4 dose requirement. The data are somewhat inconsistent. On one hand, Fierabracci et al. reported a drop in BMI and a significant downward adjustment in the mean total dose of LT4 after surgery [56], and Richou et al. observed a fall in the LT4 dose required to achieve the target TSH level after sleeve gastrectomy [57]. On the other hand, Pedro et al. did not find a difference between restrictive procedures (sleeve gastrectomy or adjustable gastric banding) and malabsorptive procedures (Roux-en-Y gastric bypass) [58], and Rubio et al. did not see a decrease (only a slight delay) in the amount of LT4 absorption after Roux-en-Y gastric bypass surgery [59]. Lastly, Fallahi et al. reported that hypothyroid patients show higher TSH levels after Roux-en-Y gastric bypass or a biliary pancreatic diversion – presumably due to poor LT4 absorption [60].

Again, the “bariatric surgery” factor might interact with the “LT4 formulation” factor: according to Fallahi et al., a switch from a tablet LT4 formulation to a liquid LT4 formulation (at the same dosage) in bariatric surgery patients was associated with a significant fall in the TSH level [60].

#### Behavioural factors

Here, we consider that behavioural factors included the voluntary ingestion of drugs, foods, and food supplements. As mentioned above, the general presence of food in the stomach (i.e. food intake in the three hours before oral LT4 administration) reduces the LT4 absorption. Furthermore, a high proportion of LT4-treated patients ingest medications, foodstuffs and food supplements that selectively or specifically reduce the intestinal absorption of LT4. These substances include over-the-counter medications (mainly antacids or acid reducers), dietary supplements (primarily calcium and iron salts), foods/beverages high in fibre, iodine or soy, proton pump inhibitors (PPIs), sucralfate, aluminium hydroxide, magnesium hydroxide, calcium salts, ferrous sulphate, raloxifene, cholestyramine, colestipol, lanthanum carbonate, cation exchange resins, and sevelamer [48, 61–63][48], some of which are described in more detail below.

The ingestion of calcium carbonate supplements is associated with lower total T4 (TT4) levels and elevated TSH levels, relative to baseline [65–67]. Indeed, LT4 can bind to calcium carbonate – potentially reducing the bioavailability [66]. After total or completion thyroidectomy for benign disease, the time needed to achieve euthyroid status (usually 3–4 months) was considerably longer and required more dose adjustments in patients taking calcium supplements [64]. Again, liquid formulations of LT4 may be less subject to interference by calcium and iron supplements [39].

Dietary fibre binds LT4 and reduces the drug’s bioavailability in a non-specific manner; in a case series reported by Liel et al. withdrawal of fibre (mainly wholewheat and fibre-enriched bread) was associated with marked reductions in TSH levels and the LT4 dose requirement [68]. However, dietary fibre has many health benefits and should necessarily not be withdrawn; hence, an LT4 dose increase can be considered.

With regard to resins and binders, concomitant administration of LT4 and the non-absorbed, potassium-binding polymer patiromer was found to decrease the area under the curve (AUC) extrapolated to infinity and the peak concentration (C_max_) for T4, according to the results of an open-label crossover study. The interaction was avoided by delaying the patiromer administration by 4 h [69]. Likewise, concomitant intake of the phosphate binder sevelamer hydrochloride significantly decreased the area under the serum TT4 concentration curve [70].

Some antibiotics also interfere with LT4 absorption: in a pharmacokinetic study of healthy volunteers taking a dose of LT4, Goldberg et al. showed that concomitant administration of ciprofloxacin was associated with a significantly lower area under the plasma T4 concentration–time curve [71].

Drugs that modify the stomach’s acidity have also been linked to LT4 malabsorption, although the effect is subject to debate. There is a large body of evidence (from general practice patient databases, electronic medical record reviews, and clinical trials) to indicate that PPIs are prominent interfering substances that raise TSH levels and/or the LT4 dose requirement [63, 72, 73]. In contrast, various clinical studies of one week of treatment with pantoprazole [74], 3 months of treatment with omeprazole [75], and a week’s treatment with esomeprazole [76] did not find significant intergroup differences in the TSH or thyroid hormone levels. We suggest that these apparent disparities in the data might be due to (i) the possible occurrence of interactions with LT4 in a subset of patients only, and/or (ii) a lack of power in some of the randomized studies cited above.

Coffee is a beverage associated with breakfast in many cultures but the strength, amount and composition varies. In Benvenga et al. pharmacokinetic study, taking LT4 with expresso Italian coffee was associated with a lower peak and a lower AUC for serum T4, and failure to achieve target TSH levels. [77]. In contrast, an acute loading test in two patients found that coffee did not modify the pharmacokinetics of LT4 [78]. Lastly, Cappelli et al. reported that a move to taking the LT4 30 min before breakfast for 3 and 6 months did not significantly modify the serum TSH level [79]. Hence, coffee (caffeine) per se does not appear to perturb LT4 uptake.

In Chon et al. pharmacokinetic study of cold or hot milk (another beverage frequently consumed at breakfast), co-administration of LT4 and 2% milk by healthy euthyroid subjects was associated with a significantly lower peak concentration (by around 8%) and a significantly lower area under the curve for TT4 (10%) [80]. However, in a patient population (infants with congenital hypothyroidism), intake of soy formula milk was associated with TSH elevation and a longer time to TSH normalization [81]. Taking LT4 with grapefruit juice was associated with statistically significant decreases (of around 10%) in the peak T4 concentration and the AUC for T4. However, TSH levels did not appear to be affected, and the investigators considered that concomitant grapefruit juice and LT4 intake did not have a clinically relevant effect [82]. It has been suggested that the “grapefruit juice effect” on the absorption of some drugs is due to inhibition of the organic anion transporting polypeptide 1A2 (OATP1A2) transporter in the intestinal mucosa, as has also been suggested for the drug ciprofloxacin [83, 84]. Nevertheless, it should be borne in mind that milk and grapefruit juice will respectively increase and decrease the pH of the stomach, which may introduce a confounding effect.

Although a large number of compounds and pathologies have been suggested as modulators of intestinal absorption of LT4, the biochemical mechanisms remain poorly characterized. In an *in vitro* study, Zu Schwabedissen et al. sought to determine whether the organic anion transporting polypeptide 2B1 (OATP2B1) was involved in the intestinal absorption of thyroid hormones [85]. The results of competitive counter-flow experiments indicated that T4 and T3 (but not rT3 or T4-gluconuride) were indeed substrates for OATP2B1. The researchers also determined (using PCR gene expression assays and Western blots) that potentially physiological micromolar concentrations of T3 and (to a lesser extent) T4 induced OATP2B1 and DIO1 gene and protein expression in some cell lines. Lastly, Kharrazian et al. found that dozens of raw or cooked food products showed immune cross-reactivity with thyroid hormones and enzymes; however, the putative effect of anti-hormone antibodies on T4 levels remains to be determined [86].

**Fig. 3:**
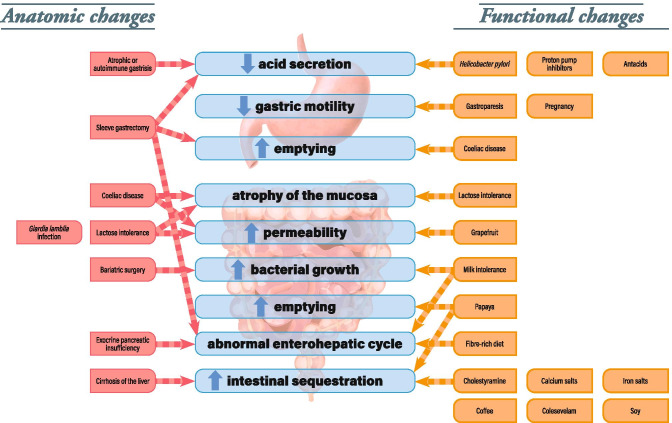
A summary of anatomic and functional changes that can reduce or slow the absorption of LT4 in the gastrointestinal tract

As mentioned in the section on the time of day of LT4 administration, taking LT4 on an empty stomach rules out a large number of variability factors and is advisable in cases of suspected malabsorption. Factors that reportedly reduce or slow the absorption of LT4 are listed in Table 2 and summarized in Fig. 3.

**Table 2:** Factors that modulate the absorption of LT4. (**↓** = decrease, **↑** = increase, **↔** = no change)

Factor	Category	Subcategory	Effect	*Suggested clinical strategy*	References
Ulcerative colitis	Pathophysiological	pathological	**↑** in the LT4 dose required to achieve the target TSH level	*Screen for ulcerative colitis*	[49]
Coeliac disease	Pathophysiological	pathological	**↑** in the LT4 dose required to achieve the target TSH level	*Screen for coeliac disease*	[50]
Bariatric surgery	Pathophysiological	pathological	**↓** in BMI, **↓** in the LT4 dose but **↓** in LT4 absorption, **↑** in the time to serum T4 peak, **↑** in mean steady-state TSH levels	*Monitor TSH and thyroid hormone levels periodically during weight loss and adjust as required*	[56–60]
Autoimmune gastritis	Pathophysiological	pathological	**↑** in the LT4 dose required to achieve the target TSH level	*Screen for serum anti-parietal cell antibodies*	[53]
*Helicobacter pylori* infection	Pathophysiological	pathological	**↑** in the LT4 dose required to achieve the target TSH level	*Screen for a Helicobacter pylori infection and eradicate if present. Consider a switch to a liquid LT4*	[43, 52, 73]
Proton pump inhibitors (e.g. omeprazole, lansoprazole, esomeprazole, and pantoprazole)	Behavioral	medications	Effect subject to debate: **↑** in TSH levels reported in studies of omeprazole and lansoprazole but no significant effect or no consensus for esomeprazole and pantoprazole	*Check for PPI treatment, check the TSH level, and increase the dose level of LT4 if TSH is elevated*	[72–76]
Tyrosine kinase inhibitors (e.g. imatinib and sorafenib)	Behavioral	medications	**↑** in TSH levels with imatinib **↓** in serum FT4 and FT3 with sorafenib, after adjustment for LT4 dose and bodyweight. **↓** in T3/T4 and T3/rT3, possibly due to an increase in DIO3 activity	*Check for the use of tyrosine kinase inhibitors; if so, increase the LT4 dose level*	[113, 115]
Alendronate	Behavioral	medications	**↔** no significant effect or no consensus	*An effervescent formulation of alendronate supplement is unlikely to interfere with concomitant LT4 treatment*	[133]
Patiromer (potassium-binding resin)	Behavioral	medications	**↓** in LT4 absorption	*Delay patiromer administration by several hours in patients taking LT4*	[69]
Ciprofloxacin (antibiotic)	Behavioral	medications	**↓** in plasma T4	*Check for concomitant use of ciprofloxacin; if so, increase the LT4 dose level*	[71]
Rifampin (antibiotic)	Behavioral	medications	**↑** in plasma T4	*Check for concomitant use of rifampin; if so, decrease the LT4 dose level*	[71, 117]
Simvastatin (statin)	Behavioral	medications	**↔ **no significant effect or no consensus	*No action required because an interaction is unlikely*	[134]
Colesevelam HCl (bile acid sequestrant)	Behavioral	medications	**↓** in serum T4	*Check for the use of the bile acid sequestrant colesevelam; if so, increase the LT4 dose level*	[123]
Lanthanum carbonate (phosphate binder)	Behavioral	medications	**↓** in serum T4	*Check for the use of phosphate binders; if so, increase the LT4 dose level*	[123]
Sevelamer hydrochloride (phosphate binder)	Behavioral	medications	**↓** in serum T4	*Check for the use of phosphate binders; if so, increase the LT4 dose level*	[70]
Fluoxetine and sertraline (selective serotonin reuptake inhibitors)	Behavioral	medications	**↓** in serum T3 and T4	*Check for the use of the SSRIs (small effect); if so, increase the LT4 dose level*	[129]
Famotidine (H2 antihistamine, antacid)	Behavioral	medications	**↔ **no significant effect or no consensus	*No action required because an interaction is unlikely*	[76]
Oral gonadotropin (infertility treatment)	Behavioral	medications	**↔ **no significant effect or no consensus	*No action required because an interaction is unlikely*	[139]
Calcium supplements	Behavioral	dietary supplements	**↑** in TSH levels, **↓** in LT4 absorption	*Calcium carbonate supplements should be taken 6 to 8 h after LT4. Also consider a switch to a liquid LT4 formulation in patients taking calcium carbonate supplements*	[64–67]
Iron supplements	Behavioral	dietary supplements	**↑** in TSH levels, **↑** in LT4 dose adjustments required to achieve the target TSH level	*Screen for the use of iron or mineral calcium supplements*	[39]
Food ingestion (breakfast)	Behavioral	foodstuffs	**↔ **no significant negative effect or no consensus for breakfast in general but some components of the meal (listed in the lines below, e.g. cow’s milk) have effects	*Check that LT4 is taken at the same time of day, regardless of whether this is before or during breakfast. Check TSH levels more frequently in patients taking LT4 with food*	[27–31]
Grapefruit juice	Behavioral	foodstuffs	**↓** in T4 peak and AUC. **↔** for TSH levels	*Monitor TSH levels*	[82]
Coffee	Behavioral	foodstuffs	**↔ **no significant effect or no consensus	*Consider a switch to a liquid formulation if the patient persists in drinking coffee at the same time as the LT4 dose*	[77–79]
Cow’s milk	Behavioral	foodstuffs	**↓** in LT4 absorption	*Check that the patient is avoiding cow’s milk and similar substances at the time of the LT4 dose*	[80]
Soy milk (formula)	Behavioral	foodstuffs	**↑** in TSH and the time to TSH normalization	*Check that the patient is avoiding milk and similar substances at the time of the LT4 dose*	[81]
Dietary fibre (wholewheat and fiber-enriched bread)	Behavioral	foodstuffs	**↑** in TSH levels and the LT4 dose requirement	*Consider withdrawing dietary fiber*	[68]
Curcumin extract	Behavioral	foodstuffs	**↔ **no significant effect or no consensus	*A curcumin supplement is unlikely to interfere with LT4 treatment*	[132]
Vitamin C	Behavioral	foodstuffs	**↓** in TSH levels and **↑** in FT4 and TT3	*Consider supplementation with vitamin C in patients with malabsorption*	[141, 142]

*LT4* levothyroxine, *TSH* thyroid-stimulating hormone, *BMI* body mass index, *T4* thyroxine, *FT3* free liothyronine, *FT4* free thyroxine, *rT3* reverse liothyronine, *DIO3* type 3 deiodinase, *AUC* area under the curve

### Factors that interfere with the distribution of T4

#### Pharmaceutical factors

We did not find any evidence to suggest that once T4 is absorbed and crosses the epithelium, its distribution is significantly modulated by LT4-related pharmaceutical variables (formulation, dosing frequency, time of day, etc.).

#### Pathophysiological factors

It is well known that the mean distribution volume of LT4 is greater in hypothyroid patients (up to 15 L) than in euthyroid subjects (typically 11–12 L) – possibly as a result of an increase in the volume of intracellular water [18, 19].

As mentioned above, Zu Schwabedissen et al. *in vitro* cell-based study showed that T4 and T3 were substrates of the drug transporter OATP2B1. Although the researchers focused on uptake across the intestinal epithelium (i.e. absorption), OATP2B1 is also expressed in other tissues – notably the liver. Hence, variations in *OATP2B1* gene transcription and translation might alter the pharmacodynamics of thyroid hormones more broadly – including their tissue distribution [85].

#### Behavioural factors

A number of drugs modify the amount of T4 bound to plasma proteins. Firstly, therapeutic levels of phenytoin, carbamazepine, salsalate, salicylate, furosemide, fenclofenac, ethacrynic acid, and heparin induce the displacement of T4 from the binding proteins *in vitro* [87, 88]. However, give the large amounts of plasma proteins in the circulation, Burch considered that the *in vivo* effects of this displacement would be negligible [88].

In postmenopausal women taking LT4, the initiation of oestrogen therapy was associated with increases in serum TBG, TT4 and TSH, and a decrease in the serum FT4 concentration [89]. The researchers suggested that an oestrogen-induced increase in serum TBG might transiently decrease the FT4 concentration and thus reduce the entry of thyroid hormones into cells. In contrast, Shifren et al. reported that transdermal oestrogen therapy (as opposed to oral administration) exerts minimal effects on the binding proteins and then on TT4 and FT4 [90].

At this point, it makes sense to mention the effect of pregnancy on LT4, which is also mainly mediated by oestrogen. The sharp rise in oestrogen levels during the first weeks of pregnancy leads to an elevation of TBG levels and thus contributes to a greater requirement for LT4 [91].

A number of other medications and drugs of abuse reportedly increase TBG levels: selective oestrogen receptor modulators, tamoxifen, heroin, methadone, 5-fluorouracil, and mitotane [88]. Hence, patients taking these drugs (especially mitotane) may require an LT4 dose increase. Conversely, exposure to glucocorticoids, niacin, and androgens leads to a fall in TBG levels and thus a decrease in the LT4 requirement [88, 92].

Factors that reportedly modulate the distribution of LT4 are listed in Table 3.

**Table 3:** Factors that modulate the distribution, metabolism, and excretion of LT4 (**↓** = decrease, **↑** = increase)

Distribution										
Factor	Category		Subcategory		Observations/putative mechanism			*Suggested action*		References
Postmenopausal treatment with estrogen	Behavioral		medications		**↑** in serum TT4, TBG and TSH. **↓** in serum FT4, possibly due to an increase in serum TBG levels			*Increase the LT4 dose if estrogen therapy is initiated*		[89]
Activity of the drug transporter OATP2B1	Pathophysiological		physiological		**↑** in *OATP2B1* gene expression levels in an *in vitro* (cell culture) study upon exposure to thyroid hormones			*Systematic OATP2B1 genotyping is not recommended. Treat the patient on the basis of other data*		[85]
Metabolism										
Factor	Category		Subcategory		Observations/putative mechanism			*Suggested action*		References
Selenium deficiency	Pathophysiological		pathological		**↓** in FT3, **↑** in glutathione peroxidase, which competes with deiodinases for selenium			*Screen for oxidative stress*		[109]
Oxidative stress	Pathophysiological		pathological		**↓** in serum malondialdehyde levels upon treatment with LT4			*Screen for oxidative stress*		[111]
Pregnancy	Pathophysiological		physiological		**↑** in FT4 during pregnancy in women with hypothyroidism. The main determinants of FT4 were weight and age			*Adjust LT4 dose for bodyweight changes during pregnancy*		[138]
Excretion										
Factor		Category		Subcategory		Observations/putative mechanism	*Suggested action*		References	
Nephrotic syndrome		Pathophysiological		pathological		**↑** in proteinuria and the renal excretion of T4 bound to transport proteins	*Screen for nephrotic syndrome*		[118, 119]	
Bile acid sequestrants (cholestyramine, colestipol and colesevelam)		Behavioral		medications		**↑** enterohepatic recycling of T4	*Screen for the use of bile acid sequestrants*		[120, 121]	
Colesevelam		Behavioral		medications		Formation of a colesevelam-T4 complex in the stomach, with **↑** excretion in the feces	*Screen for the use of colesevelam*		[122]	
Phenobarbital, phenytoin, carbamazepine, and rifampin		Behavioral		medications		**↑** in the levels of the enzyme responsible for the glucuronidation of LT4	*Screen for the use of phenobarbital, phenytoin, carbamazepine, and rifampin*		[116, 117]	

*TT4* total thyroxine, *TBG* thyroxine-binding globulin, *TSH* thyroid-stimulating hormone, *FT4* free thyroxine, *LT4* levothyroxine, *OATP2B1* organic anion transporting polypeptide 2B1, *FT3* free liothyronine, *FT4* free thyroxine, *T4* thyroxine

### Factors that interfere with the metabolism of LT4 and T4

#### Pharmaceutical factors

We did not find any evidence to suggest that the metabolism of T4 is significantly modulated by LT4-related pharmaceutical variables (formulation, dosing frequency, time of day, etc.).

#### Pathophysiological factors

Given the deiodinases’ key roles in the metabolism and action of thyroid hormones, the genetic basis for potential differences in activity has been subject to much scrutiny – albeit with conflicting results, as described below.

With regard to DIO1, Panicker et al. suggested that the *DIO1* rs2235544 C allele was correlated with elevated FT3 and low FT4 levels in both LT4-treated patients and in the general population [93]. In contrast, significant relationships between the *DIO1* genotypes on one hand and thyroid hormone parameters (LT4 dose, FT3, FT4, TSH, etc.) were not observed by Santoro et al. [94] and Arici et al. [95].

For DIO2, Appelhof et al. studied 41 LT4-treated patients with primary autoimmune hypothyroidism before and during a randomized clinical trial of LT4 monotherapy vs. L-tri-iodothyronine (LT3) + LT4 combination therapy [96]. The researchers reasoned that well-being, neurocognitive functioning, and preference might be related to local T3 levels in the brain, which are regulated by DIO2 activity. However, the patient’s status for two DIO2 variants (DIO2-ORFa-Gly3Asp and DIO2-Thr92Ala) was not associated with thyroid hormone levels or the stated preference for combination therapy. Likewise, Santoro et al. did not find a significant relationship between *DIO2* genotypes and the patients’ T4 dose level [94]. Indeed, the absence of an association between the genotype and thyroid hormone level was confirmed in many other studies [93, 97–99]. In contrast, Castagna et al. reported that heterozygosity and homozygosity for the DIO2 Thr92Ala polymorphism were significantly associated with low FT3 levels—thus suggesting a rationale for LT4 + LT3 combination therapy [100]. This was confirmed by Cantara et al. [101]. Associations between the genotype and thyroid hormone variables were also reported by Torlontano et al. (for the *DIO2* rs225014 Ala/Ala variant) [102], Arici et al. (for the rs225014 TT and rs225015 GG genotypes) [95] and Carlé et al. (for the rs225014 polymorphism in *DIO2* and the rs17606253 polymorphism in *MCT10/SLC16A10*) [103].

Indirect effects on DIOs have also been suggested. In a study of 40 adult patients with hypothyroidism due to Hashimoto disease, Papanas et al. found that the serum interleukin (IL)-6 level was significantly and positively correlated with the LT4 dose, the LT4 dose per kg body weight, and the serum tumour necrosis factor alpha level but was significantly and negatively correlated with the serum TT3 level and the serum T3/T4 ratio [104]—suggesting that IL-6 inhibited the deiodination of T3 and rT3.

With regard to other enzymes and transporters, Santoro et al. investigated *UGT1A1* and *UGT1A3* genotypes in a group of LT4-treated patients having undergone total thyroidectomy and radioiodine therapy [94]. The T4 dose was found to be associated with the *UGT1A* haplotype; this was attributed to low *UGT1A1* expression and T4 glucuronidation in carriers of the *UGT1A1* rs8175347 allele. In a regression model, however, the *UGT1A* haplotype accounted for only 2% of the total variability in T4 dose level and was not considered to be clinically relevant. With regard to the monocarboxylate transporter 10 (MCT10, an amino acid transporter), Cantara et al. reported that patients homozygous or heterozygous for the rs17606253 polymorphism in *SLC16A10* had significantly lower FT3 levels than other individuals [101]. Roef et al. study in Belgium found that the rs5937843 (G/T) polymorphism in the *SLC16A2* gene coding for the monocarboxylate transporter 8 (MCT8, which transports T3 and T4 specifically) was inversely associated with FT4 levels in men but not in women. Similarly, the rs6647476 (T/C) polymorphism in *MCT8* was inversely associated with FT3 levels but again only in men [105]. It is noteworthy that inactivating mutations in *SLC16A2* produce a severe X-linked syndrome (Allan-Herndon-Dudley syndrome) with a lack of TH in target brain regions and excess TH in the peripheral tissues [106].

Cardiovascular disease and heart failure are associated with elevated levels of oxidative stress and the selenoprotein glutathione peroxidase, which in turn have been linked to selenium (Se) deficiency, competition for Se, and low deiodinase activity [107, 108]. Fraczek-Jucha et al. reported that serum Se and FT3 levels were not correlated even when the prevalence of Se deficiency was high (74.6%) [109]. Yang et al. single-centre study of a cohort of ambulatory patients in the US found that the dose of LT4 did not differ significantly when comparing patients with heart failure vs. those without [110]. Lastly, Chakrabarti et al. found that serum malondialdehyde levels (a marker of oxidative stress) fell during treatment with LT4 [111]. Se supplementation did not have a significant additional effect.

#### Behavioural factors

The metabolism of T4 can be significantly modulated by behavioural factors, such as medication use. Firstly, the deiodinases’ action might also be affected by various drugs or other compounds: Olker et al. *in vitro* screening study of recombinant DIOs found that 411 (22.5%) of the 1851 compounds from the ToxCast Phase 2 and e1k libraries inhibited DIO1, DIO2 and/or DIO3 by more than 20%, and that 228 (12.5%) inhibited a DIO by at least 50% [112]. The most potent inhibitors had IC_50_ values in the micromolar range, and 81 chemicals were specific inhibitors (i.e. inhibition in only one of the three deiodinases).

With regard to a putative effect of tyrosine kinase inhibitors (TKIs) on DIO3, Abdulrahman et al. found that 26 weeks of sorafenib treatment in 21 LT4-treated patients with progressive non-medullary thyroid carcinoma was associate with falls in serum FT4 and FT3 levels and the T3/T4 and T3/rT3 ratios [113]. The researchers suggested that these changes were due to a sorafenib-induced increase in DIO3 activity. An increase in DIO3 activity can also be observed in consumptive hypothyroidism syndrome (associated with tumours such as hepatic hemangioendotheliomas, gliomas, neuroblastomas, colon carcinomas, gastrointestinal stromal tumours, and fibrous tumours), gestational hypothyroidism and treatments with oestrogens. More generally, thyroid dysfunction is a well-known adverse effect of TKIs in euthyroid individuals – although not necessarily by modifying the metabolism of LT4 –and so may increase the requirement for LT4 in individuals with hypothyroidism [114, 115].

Treatments with phenobarbital, phenytoin, carbamazepine, and rifampin induce an increase in the levels of the enzyme responsible for the glucuronidation of LT4 [116, 117]. In Goldberg et al. pharmacokinetic study, for example, the concomitant administration of rifampin and LT4 was associated with a significantly higher area under the plasma T4 concentration–time curve [71].

The factors that reportedly modulate the metabolism of LT4 are summarized in Table 3.

### Factors that interfere with the excretion of LT4 and T4

#### Pharmaceutical factors

We did not find any reports on the specific modulation of T4 excretion by LT4-related pharmaceutical variables (formulation, dosing frequency, time of day, etc.).

#### Pathophysiological factors

With regard to the specific modulation of T4 excretion by pathophysiological variables, the occurrence of nephrotic syndrome with marked proteinuria may increase the renal excretion of T4 bound to transport proteins like TBG. Hence, the LT4 requirement would increase in this context [118, 119].

#### Behavioural factors

The bile acid sequestrants cholestyramine, colestipol and colesevelam decrease the T4 concentration – probably via the augmented enterohepatic recycling of T4 [120, 121], although cholestyramine additionally forms a complex with T4 in the stomach and thus increases the amount of T4 excreted in the feces [122]. The serum TT4 concentration curve is decreased by around 95% and 40% by the administration of the bile acid sequestrant colesevelam and lanthanum, respectively, relative to LT4 taken alone [123]. As mentioned above, treatments with phenobarbital, phenytoin, carbamazepine, and rifampin induce an increase in the levels of the enzyme responsible for the glucuronidation of LT4, and thus will modify (albeit indirectly) the latter’s excretion [116, 117].

Factors that reportedly modulate the excretion of LT4 are summarized in Table 3.

Lastly, factors that reportedly modulate the effectiveness of LT4 but whose mechanisms are unknown are listed in Table 4 [124].

**Table 4:** Other factors that modulate the effectiveness of LT4 (mechanisms unknown). (**↓** = decrease, **↑** = increase)

Factor	Category	Subcategory	Observations/putative mechanism	*Suggested action*	References
Female sex	Pathophysiological	physiological	**↑** Changes in estrogen levels during events such as pregnancy and menopause might decrease the FT4 concentration and thus reduce the entry of thyroid hormones into cells	*Monitor the LT4 maintenance dose in female patients as a function of life events*	[89–91]
Kidney transplantation	Pathophysiological	pathological	Kidney transplantation was associated with a marked fall in the required dose of LT4. Patients who subsequently return to dialysis required an LT4 dose increase	*Decrease the LT4 dose after kidney transplantation*	[124]
Food antigens	Pathophysiological	foodstuffs	**↑** in immune cross-reactivity between thyroid hormones/enzymes and raw or cooked food products, as a possible trigger for thyroid disorders	*Consider repeating the thyroid hormone assays with kits/techniques*	[86]
Zinc	Pathophysiological	foodstuffs	**↑** in serum FT3 after supplementation with Zn (alone or combined with Se)	*Consider zinc supplementation in overweight or obese patients with hypothyroidism*	[135]

*FT3* free liothyronine, *FT4* free thyroxine, *LT4* levothyroxine, *T4* thyroxine

### Factors that do not appear to worsen the ADME parameters of LT4

Many drugs and foods appear to have no effect on LT4 or an effect that is subject to debate. In a study of eight obese, diabetic postmenopausal women with primary hypothyroidism, Isidro et al. found that after 3 months of treatment with metformin, the mean ± SD TSH level (1.18 ± 0.36 µIU/ml) was significantly lower than at baseline (3.11 ± 0.50 µIU/ml). The mean FT4 level was slightly but not significantly higher [125]. Metformin treatment has a TSH-lowering effect in patients with LT4-treated or untreated polycystic ovary syndrome or in LT4-treated patients with diabetes mellitus [126, 127]. The TSH-lowering effect in untreated patients (i.e. those not taking LT4) means that the metformin is unlikely to act by modifying LT4 absorption. Animal studies have also shown that the low TSH levels are not due to a metformin-induced increase in LT4 absorption [128]. Hence, metformin’s effect (or lack of effect) on TSH and FT4 levels requires further investigation.

De Carvalho et al. performed a prospective, controlled study of fluoxetine or sertraline initiation in 28 patients with major depression and hypothyroidism and 29 patients with major depression and normal thyroid function. Patients on fluoxetine experienced a significant fall in the TT3 level after 15 and 30 days of treatment and the TT4 levels after 15, 30 and 90 days, although the values remained in the euthyroid range. Sertraline had similar but weaker effects [129]. Other substrates that do not appear to influence the rate and extent of LT4 absorption and its effects include soy isoflavones [130], the antacid famotidine, the lipid-lowering compound ezetimibe [76], the anticoagulant warfarin [131], a curcumin supplement for osteoarthritis [132], the osteoporosis drug alendronate [133], and simvastatin [134]. Interestingly, supplementation with Zn (alone or combined with Se) for 12 weeks was associated with a significant increase in serum FT3 levels but had no significant effects on TT3, FT4, TT4 or TSH levels [135]. Although Zn is known to interact with thyroid hormone systems in a complex manner, it is not clear whether Zn supplementation influences deiodinase levels or activity [136].

Women with hypothyroidism are generally advised to increase the dose of LT4 by 30 to 50% (depending on the residual endogenous thyroid function) because of weight gain, a larger T4 pool (an elevated TBG level), transfer to the foetus, and increased clearance of T4 (due to placental DIO3 activity) [137]. Haddow et al. found that FT4 levels were higher in pregnant women treated for hypothyroidism than in pregnant euthyroid women with equivalent TSH levels, as is also the case when comparing non-pregnant women treated for hypothyroidism and non-pregnant euthyroid women with equivalent TSH levels; hence, pregnancy per se did not change the high FT4 levels associated with LT4 treatment [138]. Davis et al. compared TSH levels in 5 hypothyroid women who conceived naturally and 4 who conceived after gonadotropin stimulation or with oral medications for ovulation induction. The post-conception TSH level and LT4 doses did not differ significantly when comparing the two group. The researchers concluded that fertility treatment did not require additional LT4, relative to that recommended for natural pregnancy [137, 139]. Indeed, in real life, it is necessary to monitor thyroid function (TSH) in LT4-treated women participating in an assisted reproductive technology program because the associated, transient, high oestrogen level might increase the LT4 requirement [140].

Some substances may even improve LT4 absorption. For example, Antunez and Licht found that after 2 months of co-administration of LT4 tablets with 1 g of vitamin C, the patients’ TSH levels had fallen by an average of around 70% for the same LT4 dose [141]. The effect of vitamin C was confirmed by Jubiz and Ramirez in a longitudinal study of 31 patients [142]. The researchers suggested that vitamin C might increase the solubility of LT4 in the stomach [142]. Thus, vitamin C is the only nutrient positively associated with LT4 absorption and can thus be proposed as a co-adjuvant in patients with gastrointestinal pathologies and LT4 malabsorption.

## Discussion

**Fig. 4:**
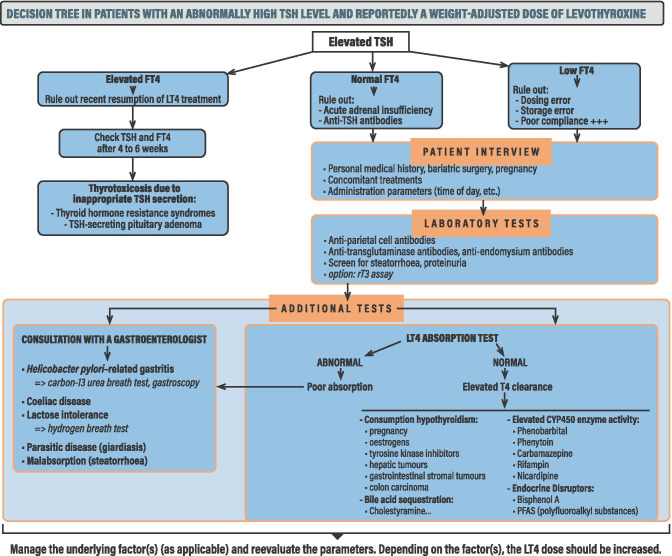
A decision tree for patients with an abnormally high TSH level but who are reportedly taking the recommended, weight-adjusted dose of LT4

Our review of the literature enabled us to draw up an extensive list of pharmaceutical, pathophysiological and behavioural factors that influence the absorption, distribution, metabolism and excretion of LT4. The great majority of these factors affected the absorption or (to a lesser extent) the metabolism (Fig. 4). When the physician is faced with a case of an abnormally high TSH level in a patient reportedly taking the recommended, weight-adjusted dose of LT4 (greater than 2 µg/kg/day or high dose to obtain and maintain a normal TSH concentration), we recommend considering the aspects summarized in Fig. 4.

The first consideration is to assay the serum FT4 level. An elevated serum FT4 level may indicate the recent resumption (in the previous week) of LT4 treatment or an LT4 dose increase or the T4 administration 60–90 min before the blood withdrawal; normally, the physician will be aware of these events and can review the patient’s TSH and FT4 levels 4 to 6 weeks later. Thyroid hormone resistance syndromes are rare but can be considered once the patient’s endocrine profile is well documented; to gain a fuller clinical picture and rule out hereditary factors, it may be worth measuring TSH and FT4 levels in the patient’s family members [143]. Autonomous TSH secretion by a TSH-secreting pituitary adenoma can also be considered [144]. Lastly, patients with congenital hypothyroidism (due to GLIS3 mutations, for example) can acquire resistance to the action of thyroid hormones [145]. A normal serum FT4 level should prompt the physician to check for acute adrenal insufficiency (especially in patients treated for chronic and primary adrenal disease) or the presence of heterophilic anti-TSH antibodies. Lastly, a low serum FT4 level may be related to a dosing error, an LT4 storage problem, LT4 malabsorption, or pseudo-malabsorption (i.e. poor compliance with LT4 treatment).

For patients with a normal or low serum FT4 level and an elevated TSH level, the next step is a detailed interview in which the patient is questioned about his/her personal and family medical histories (notably any previous surgical operations involving the digestive tract), pregnancy, and medication habits. In particular the patients should be asked to describe how and when he/she takes the LT4 (the time of day, before/during/after meals, with a drink other than water, etc.) and any other source of variability. A medication review (i.e. the systematic appraisal of all aspects of a patient’s medication management, with a view to optimization and better outcomes) may be of value – especially in older adults who might not recall the full list of their medications in an interview [146]. A possible decrease in residual thyroid function (in patients with autoimmune thyroiditis) should be considered.

If the patient interview has not revealed an obvious factor for a normal or low LT4 concentration, several laboratory tests can be prescribed. Those will include a screen for antibodies against gastric components (e.g. circulating IgGs against parietal cells [54] and intestinal antibodies against endomysium or tissue transglutaminase 2 as specific markers of coeliac disease [147]) and for *Helicobacter pylori* infection. An abnormally high level of rT3 (an analyte not assayed on a routine bases) suggests that conversion of T4 to rT3 by DIO3 is favored over the production of T3 in the context of consumptive hypothyroidism [148]. The feces can be screened for steatorrhoea (rare in hypothyroid patients) as a sign of small intestine disease and possible malabsorption. Urine dipsticks can be used to screen for a nephrotic syndrome with proteinuria.

If the clinical problem has not been revealed by the patient interview and/or laboratory assays, the next step is performance of a pharmacodynamic LT4 absorption test (LAT). Although many different protocols have been used, a LAT typically involves the oral administration (under medical supervision) of LT4 with water in the morning after fasting for at least 8 h. Venous blood samples are then taken 1, 2, 3, 4, 5 and 6 h after the LT4 and are assayed for T4. However, the lack of standardization means that LAT parameters vary from one centre to another: the acute oral LT4 dose (a fixed dose of 1000 µg vs. the patient’s normal daily dose), the measurement of TT4 or FT4, the equation used to calculate absorption, the threshold for a normal result (typically 60% or 65% of the administered dose at the 3 h time point), and the list of contraindications (mainly ischemic heart disease) [24, 149–152].

A positive LT4 absorption test result (i.e. normal absorption) is suggestive of the patient-related problems mentioned above: a dosing error, a storage error, and/or poor compliance. However, there are several other possible explanations for an normal LT4 absorption test result: (i) an increase in the digestive excretion of T4 via stimulation of the enterohepatic cycle (e.g. by cholestyramine), (ii) an increase in DIO3 activity, (iv) the effects of medications (such as phenytoin, phenobarbital, rifampin, and nicardipine), or (v) possible consequences of endocrine disruptors (bisphenol A, and per/polyfluoroalkyl compounds) [153].

In fact, the endocrine disruptors bisphenol A (2,2-bis(4-hydroxyphenyl)propane) and other bisphenols (such as bisphenol B) are structurally similar to T4 and T3 and now known to have several effects on the thyroid hormone system [154, 155]. In addition to antagonizing nuclear thyroid receptors and thus interfering with hormone-stimulated transcriptional activity, bisphenols also appear to directly influence gene expression in the thyroid and pituitary gland [156]. Furthermore, da Silva et al. reported that bisphenol A inhibits the activities of DIO1 and DIO2 *in vitro* and (when administered orally to adult male Wistar rats) is associated with significantly lower hepatic DIO1 activity (but not brown adipose tissue DIO2 activity) [157]. Serum T4 levels were abnormally high in rats treated with bisphenol A, while T3 remained unchanged [157]. Although hydroxylated and halogenated bisphenols bind competitively to thyroid hormone transport proteins like TBG and transthyretin [158], the low binding constants (relative to T3 and T4) and low bisphenol concentrations *in vivo* might not have a major influence on transport in LT4-treated patients [159].

An abnormally slow or low increase in T4 levels in the absorption test should prompt a consultation with a gastroenterologist, who will typically screen for *H. pylori*-related gastritis (using the carbon-13 urea breath test), autoimmune gastritis (a test for anti-parietal cell antibodies, plus a gastroscopic assessment of the atrophy of gastric mucosa), coeliac disease, lactose intolerance (using the hydrogen breath test), parasitic disease (giardiasis), and steatorrhoea (which increases the fecal loss of LT4).

If true LT4 malabsorption has been ruled out, the physician can consider a diagnosis of pseudo**-**malabsorption. Rather than poor LT4 storage or a poor dosing technique, the primary cause for pseudo-malabsorption is likely to be poor compliance for psychological reasons. Indeed, this poor compliance might not be readily acknowledged by the patient. Lips et al. have recommended a subtle, non-confrontational approach to solving the problem of pseudo-malabsorption [160]. The physician should explain the treatment of hypothyroidism, emphasize the benefits of good compliance and, conversely, highlight the harmful consequences of chronic hypothyroidism and therefore of poor compliance. If this subtle approach does not resolve the problem, other possible approaches include changes in the pharmaceutical form for oral administration, weekly oral ingestion of LT4 under the supervision of medical staff, or exceptionally (and preferably at an expert centre) the parenteral infusion of LT4.

Our literature search did not identify any publications on interactions between bacterial or viral infections and the effectiveness of LT4. The initial data collected during the pandemic of coronavirus 2019 disease suggest that treated hypothyroidism is neither a risk factor nor a severity factor for infection by severe acute respiratory syndrome coronavirus 2 (SARS-CoV-2) [161]. Nevertheless, it has been suggested that thyroid status should be reassessed in euthyroid pregnant women infected by SARS-CoV-2 in their first trimester of pregnancy, notably if they are at high risk of thyroid dysfunction or have a history of thyroid autoimmune disease [162, 163].

The present study had a number of limitations. Firstly, only the PubMed database was searched. Secondly, only publications in English were screened.

Perspectives for further research include the broader application of in silico simulations of oral drug absorption and the effect of drug interactions [164]. There is also scope for better evaluating the risk of malabsorption in newly treated patients, using rapid screening questionnaires. For example, Bellastella et al. recently developed and tested a seven-question “Evaluation of Malabsorption in Patients with Hypothyroidism” (EMPATHY) questionnaire [165]. The questions covered gastritis, gastroesophageal reflux disease, *H. pylori* infection, bowel disease, food allergies, dietary habits linked to a high soy intake, alcohol intolerance or addiction, nickel allergy, and intolerance of gluten, histamine, lactose, citric acid or corn starch. Use of the EMPATHY questionnaire was associated with a lower requirement for subsequent dose adjustments.

## Data Availability

The datasets (search query and list of publications) are presented in the main manuscript.

## Abbreviations

ADME: Absorption, distribution, metabolism and excretion
AUC: Area under the curve
BMI: Body mass index
EMPATHY: Evaluation of malabsorption in patients with hypothyroidism
DIO1: Type 1 deiodinase
DIO2: Type 2 deiodinase
DIO3: Type 3 deiodinase
FT3: Free T3
FT4: Free T4
LT4: Levothyroxine
LAT: LT4 absorption test
T3: L-tri-iodothyronine
MCT: Monocarboxylate transporter
OATP1A2: Organic anion transporting polypeptide 1A2
OATP2B1: Organic anion transporting polypeptide 2B1
Cmax: Peak concentration
PPI: Proton pump inhibitor
rT3: Reverse T3
SARS-CoV-2: Severe acute respiratory syndrome coronavirus 2
SNP: Single nucleotide polymorphism
SD: Standard deviation
TH: Thyroid hormone
TKI: Tyrosine kinase inhibitor
TSH: Thyroid-stimulating hormone
TBG: Thyroxine-binding globulin
TT3: Total T3
TT4: Total T4
